# RELATIONSHIP BETWEEN ESOPHAGITIS GRADES AND ***HELICOBACTER PYLORI***

**DOI:** 10.1590/0102-6720201600030002

**Published:** 2016

**Authors:** Patrícia Fernanda Saboya RIBEIRO, Luiz Fernandao KUBRUSLY, Paulo Afonso Nunes NASSIF, Irma Cláudia Saboya RIBEIRO, Andressa de Souza BERTOLDI, Venessa Caroline BATISTÃO

**Affiliations:** 1 Postgraduate Program in Principles of Surgery, Evangelic Faculty of Paraná/University Evangelic Hospital of Curitiba/Medical Research Institute, Curitiba, PR, Brazil; 2 Gastrointestinal Endoscopy Service, 9 of July Hospital, São Paulo, SP, Brazil

**Keywords:** Endoscopy, Esophagus, Esophagitis, Helicobacter pylori

## Abstract

**Background:**

The Helicobacter pylori infection (HP) is related to the development of gastric lesions and lymphoma; however, it is not known if there is a relation with gastroesophageal reflux disease and reflux esophagitis.

**Aim:**

To evaluate HP's relationship with esophagitis in patients undergoing upper endoscopy.

**Methods:**

Observational, retrospective and cross-sectional study, being evaluated 9576 patients undergoing outpatient endoscopic examination during the period between January and December 2015. Were included patients with any esophageal alteration at the examination; greater than 18; of both genders; independent of the complaint or the reason for the examination, illness or drug use. Were excluded those with active bleeding during the examination and in use of anticoagulants. The variables gender, age, esophagitis and result of the urease test, were studied. For statistical analysis was used the Epi Info software 7.1.5.2.

**Results:**

Most of the samples consisted of women and the overall average age was 46.54±16.32 years. The presence of infection was balanced for gender: 1204 (12.56%) women and 952 (13.92%) men. Relating degree of esophagitis HP- and HP+ was observed that the type A was the most common (58.79%, n=1460); 604 (24.32%) had grade B; 334 (13.45%) grade C, and 85 (3.42%) grade D. In the relation between the grade of esophagitis with gender, esophagitis A was predominant in women and present in 929 (63.33%), followed by type B, 282 (46.68%), 136 C (40.71%) and D 30 (35.29%). In men 531 (36.36%) showed type A, 322 (53.31%) B, 198 (59.28%) C, and 55 (64.70%) D. Among the groups 40-50 and over 60 years there was a significant difference in whether have or not have HP+.

**Conclusion:**

There is no significant difference between HP infection and the different grades of esophagitis.

## RESUMO

**Racional:**

A infecção pelo Helicobacter pylori (HP) é relacionada com o desenvolvimento de lesões e linfoma gástricos; porém, ainda não se sabe ao certo se há relação dele com a doença do refluxo gastroesofágico e esofagite de refluxo.

**Objetivo:**

Avaliar a relação do HP com as esofagites em pacientes submetidos à endoscopia digestiva alta.

**Métodos:**

Estudo observacional, retrospectivo e transversal, sendo avaliados 9576 pacientes submetidos ao exame endoscópico ambulatorial durante o período compreendido entre janeiro e dezembro de 2015. Foram incluídos pacientes que apresentaram alguma alteração esofágica ao exame; maiores que 18 anos; de ambos os gêneros; independente da queixa ou da razão para a realização do exame, doença ou uso de medicamentos. Excluíram-se os com sangramento ativo durante o exame e em uso de anticoagulantes. Foram avaliadas as variáveis gênero, idade, esofagite e resultado do teste da urease. Para a análise estatística utilizou-se o software Epi Info 7.1.5.2.

**Resultados:**

A maioria das amostras foi composta por mulheres e a idade média geral foi de 46,54±16,32 anos. A presença da infecção foi equilibrada para a variável gênero: 1204 (12,56%) mulheres e 952 (13,92%) homens. Relacionando os graus da esofagite com HP+ e HP- observou-se que o tipo A foi o mais comum (58,79% da amostra, n=1460), que 604 (24,32%) possuíam o grau B; 334 (13,45%) o grau C e 85 (3,42%) o grau D. Já na relação entre os graus de esofagite com o gênero, a esofagite A foi predominante nas mulheres e presente em 929 (63,33%), seguido pelo tipo B, com 282 (46,68%), C com 136 (40,71%) e D com 30 (35,29%) mulheres. Nos homens 531 (36,36%) apresentam o tipo A, 322 (53,31%) o B, 198 (59,28%) o C e 55 (64,70%) o D. Entre os grupos de 40 a 50 anos e acima de 60 anos houve diferença significativa em ter ou não HP+.

**Conclusão:**

Não há diferença significativa entre infecção por HP nos diferentes graus de esofagite.

## INTRODUCTION

Infection by *Helicobacter pylori* (HP) is associated with the development of lymphoma and gastric lesions; however, it is not known for sure if there is a relationship with gastroesophageal reflux disease (GERD) and reflux esophagitis^1^ ^,^ ^12^ ^,^ ^17^. It is believed that its action in reflux esophagitis is due to three mechanisms: increase the predisposition to GERD by increased acid secretion and decreased esophageal sphincter pressure; by its direct action in the esophageal epithelium; and indirectly by the action of toxic substances secreted by the body due gastric reflux^2^ ^,^ ^10^ ^,^ ^11^.

The incidence of infection by the bacteria in patients with GERD is variable in the literature, between 30-90%, and 35% in most series, probably by the geographic particularities ^3^.

It was observed that the decrease in the prevalence of HP was accompanied by an increase in the incidence of GERD and its complications^14^. Nevertheless, the relationship between them is uncertain as well as eradication effects on GERD.

The aim of this study was to evaluate HP's relationship with esophagitis in patients undergoing upper endoscopy.

## METHOD

This is an observational, retrospective and cross study. Were evaluated 9576 patients undergoing outpatient endoscopic examination during the period between January and December 2015.

Inclusion criteria were patients who presented any amendment to esophageal examination; greater than 18; of both genders; independent of the complaint or the reason for the examination, illness or drug use. Were excluded those with active bleeding during the examination and use of anticoagulants. Gender, age, esophagitis and result of the urease test were evaluated.

The patients were previously submitted to the usual preparation for endoscopy: fasting for 8 h for solids and liquids. Immediately before the test were asked to ingest 10 ml of water with 40 drops of simethicone and sprayed the oropharynx with lidocaine spray 5-10 puffs. All tests were performed in the presence of a second doctor in the room responsible for sedation.

Endoscopic examinations were performed according to the conventional technique with videoscopes devices (Fujinon(r)) by different members at the Endoscopy Unit Digestive, Diagnostic Center and Endoscopic Therapy of São Paulo, July 9 Hospital, São Paulo, SP, Brazil, with standardization of diagnostics and internal quality control. The endoscopic diagnoses included focused on the different degrees of erosive esophagitis - A, B, C, and D Los Angeles classification.

For the urease test were carried out three biopsies in all patients: distal body, at incisura angularis and in the antrum, and performed with biopsy forceps. The material was immediately placed on the bottle with the prefabricated reagent. It was expected 2 h for reading the test result.

The statistical analysis used the statistical software Epi Info 7.1.5.2. 

## RESULTS

Were collected 9576 cases during the period and from these, 2483 patients with esophagitis were selected. The majority was women (61.2%) and the overall average age was 46.54±16.32 years. The urease test was negative in most cases (n=2156, 86.83%) and positive in 327 (13.16%). The sample with positive urease was balanced for gender: 12.56% women and 13.92% men, with no significant difference (p=0.208), ie, there was no influence of gender on the outcome.

Considering the degree of esophagitis and its relationship with HP and HP-+ (Table 1) it is observed that grade A was the most common (58.79%, n=1460); 604 (24.32%) were grade B; 334 (13.45%) C, and 85 (3.42%) D.

**TABLE 1:** Esophagitis grades and its relationship with HP+ and HP-

Esophagitis	H. pylori +	%	H. pylori -	%	Total
A	214	15.65	1246	85.34	1460
B	70	11.58	534	88.41	604
C	37	11.07	297	88.92	334
D	6	7.05	79	92.94	85
Total	327		2156		2483

In the relationship between esophagitis grades with gender (Table 2) esophagitis A was predominant in women, present in 929 (63.33%) patients, followed by grade B with 282 (46.68%), C with 136 (40.71%) and D with 30 (35.29%). In men, 531 (36.36%) were in grade A, 322 (53.31%) in B, 198 (59.28%) in C and 55 (64.70%) in D.

**TABLE 2:** Esophagitis distribution in relation to gender

Esophagitis	Women	%	Men	%	Total
A	929	63.63	531	36.36	1460
B	282	46.68	322	53.31	604
C	136	40.71	198	59.28	334
D	30	35.29	55	64.70	85
Total	1377		1106		2483

With respect to age (Table 3), the frequency of esophagitis occurred in all age groups, with peak incidence around 60 years. The frequency between 10-20 years was much lower than that observed in the other groups.

**TABLE 3:** Different grades of esophagitis and its distribution by age

Age group	Esophagitis A	Esophagitis B	Esophagitis C	Esophagitis D	Total
< 10	1	0	2	0	3
10-20	46	12	0	0	58
20-30	238	82	26	11	357
30-40	292	136	65	10	503
40-50	277	102	52	10	441
50-60	307	151	89	22	569
60-70	203	90	56	6	355
70-80	80	24	29	16	149
>80	16	7	15	10	48
Total	1460	604	334	85	2483

In the grouped analysis for age (Table 4), that measures the age influence in the incidence of infection p was significant (p<0.05) between groups 1 and 2, 1 and 3, 1 and 4, 4 and 6. The frequency observed in group 1 (10-20) is much lower than that observed in the other groups, with statistical difference. Among the groups 4 (40- 50) and 6 (over 60 years) there was also a significant difference, whether or not HP+; group 4 was more likely to have HP infection than the group 6.

**TABLE 4:**
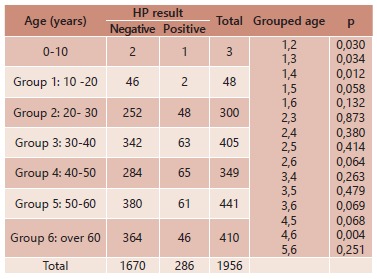
Cross grouped tabulation by age and HP

## DISCUSSION

The literature presents conflicting results regarding the influence of HP in the development of GERD and esophagitis. Some studies suggest that its eradication may be associated with the development of reflux esophagitis and it has been proposed that individuals infected with the positive CagA strain had decreased risk of GERD and its complications^6^, while others have shown that the symptoms of heartburn improve after eradication of HP and there would be no increased incidence of GERD and esophagitis^7^ ^,^ ^8^ ^,^ ^15^ ^,^ ^18^. In any event, it is important to evaluate the cost/benefit of treatment since infection by the bacterium is known to be associated with other diseases, such as gastric cancer.

The apparent protective effect of HP in GERD seems to be associated with the type of its gastric injury. Those with predominant gastritis in antrum have gastric acid hypersecretion, while pangastritis or predominant gastritis in the body have reduced acid secretion. Decreased gastric acidity with consequent increase in gastrin, increasing the lower esophageal sphincter pressure, may explain the inverse relationship between HP infection and DRGE^13^.

Ronkainen et al.^13^ studying the relationship between eosinophilic esophagitis and HP infection, found 48 patients with this type of esophagitis, eight of whom were infected. Four were clearly classified as eosinophilic esophagitis and correlation with HP had OR=0.41 suggesting an inverse relationship between infection and this type of esophagitis. There is no data on this inverse relationship between the bacteria and other non-allergic esophagitis^17^.

Some studies have shown that male gender is predictive for the presence of esophagitis^4^ ^,^ ^5^ ^,^ ^6^. In this study was found esophagitis more often in women than in men, but with no significant difference. The infection in this study was relatively balanced for gender with positive urease, although most of the sample was composed by women. Thus, despite the similar infection rate, more women had esophagitis in this sample, but without significant difference.

Raquel^16^ noted that the prevalence of HP infection in 250 individuals was not significantly different between the groups with erosive esophagitis and without. The bacteria was found in 74 (77%) and 120 (78%) subjects in each group. Furthermore, it related erosive esophagitis in most cases (73.4%) of grades l and ll Savary-Miller. Although the group with erosive esophagitis serologic prevalence of positive antiCagA was lower (74%) than without esophagitis (83%) and even lower in individuals with more severe esophagitis (67%), the values ​​found were not statistically significant and it was concluded that the presence or severity of erosive esophagitis are not associated with gastric HP serology anti-CagA positive or negative infection.

There was association between inflammatory findings and the HP results, obtaining the value 8.1993 and p=0.0421 for probability higher than the significance level defined for the test (α=0.05); therefore, it can be concluded that for this sample, there was no influence of the presence of bacteria and esophagitis.

 There was esophagitis in all age groups with a peak incidence around 60 years. The analysis of the grouped rate for age resulted in significant p, lower between 10 and 20 years than in the other groups; between groups of 40 to 50 years and above 60 there was also a significant difference in whether or not HP+, prevailing greater chance of HP+ between 40 and 50 years, while literature^1^ shows that the distribution by gender and age is similar in groups with or without esophagitis.

## CONCLUSION

There is no significant difference between HP infection in different grades of esophagitis in relation to gender; however, among the 40 groups 50 years and over 60 years there is a significant difference in whether or not are HP+.
